# Brain ureido degenerative protein modifications are associated with neuroinflammation and proteinopathy in Alzheimer’s disease with cerebrovascular disease

**DOI:** 10.1186/s12974-017-0946-y

**Published:** 2017-09-02

**Authors:** Xavier Gallart-Palau, Aida Serra, Benjamin Sian Teck Lee, Xue Guo, Siu Kwan Sze

**Affiliations:** Division of Chemical Biology & BioTechnology, School of Biological Sciences, Nanyang Technological University, 60 Nanyang Drive, Singapore, 637551 Singapore

**Keywords:** Deimination, Carbamylation, Proteinopathy, Citrullination, Mixed dementias, Alzheimer’s disease

## Abstract

**Background:**

Brain degenerative protein modifications (DPMs) are associated with the apparition and progression of dementia, and at the same time, Alzheimer’s disease with cerebrovascular disease (AD + CVD) is the most prevalent form of dementia in the elder population. Thus, understanding the role(s) of brain DPMs in this dementia subtype may provide novel insight on the disease pathogenesis and may aid on the development of novel diagnostic and therapeutic tools. Two essential DPMs known to promote inflammation in several human diseases are the ureido DPMs (uDPMs) arginine citrullination and lysine carbamylation, although they have distinct enzymatic and non-enzymatic origins, respectively. Nevertheless, the implication of uDPMs in the neuropathology of dementia remains poorly understood.

**Methods:**

In this study, we use the state-of-the-art, ultracentrifugation-electrostatic repulsion hydrophilic interaction chromatography (UC-ERLIC)-coupled mass spectrometry technology to undertake a comparative characterization of uDPMs in the soluble and particulate postmortem brain fractions of subjects diagnosed with AD + CVD and age-matched controls.

**Results:**

An increase in the formation of uDPMs was observed in all the profiled AD + CVD brains. Citrulline-containing proteins were found more abundant in the soluble fraction of AD + CVD whereas homocitrulline-containing proteins were preferentially abundant in the particulate fraction of AD + CVD brains. Several dementia-specific citrulline residues were also identified in soluble proteins previously categorized as pro-immunogenic, which include the receptor P2X7, alpha-internexin, GFAP, CNP, MBP, and histones. Similarly, diverse dementia-specific homocitrulline residues were also observed in the particulate fractions of AD + CVD in proteins that have been vastly implicated in neuropathology. Intriguingly, we also found that the amino acids immediately flanking arginine residues may specifically influence the increase in protein citrullination.

**Conclusions:**

Taken together, these results indicate that uDPMs widely contribute to the pathophysiology of AD + CVD by promoting neuroinflammation and proteinopathy. Furthermore, the obtained results could help to identify disease-associated proteins that can act as potential targets for therapeutic intervention or as novel biomarkers of specific neuropathology.

**Electronic supplementary material:**

The online version of this article (10.1186/s12974-017-0946-y) contains supplementary material, which is available to authorized users.

## Background

Dementia originates from a range of neurodegenerative and proteinopathic diseases that are predicted to affect over 130 million people worldwide by 2050 [1]. Alzheimer’s disease (AD) is the leading cause of this epidemic syndrome, and it accounts for about 60% of dementia cases [2]. Cerebrovascular disease (CVD) accounts for about 20% of dementia cases [3] and often coexists with AD in AD + CVD, a mixed form of dementia that remains as the most prevalent subtype among the elder population [4].

Several protein posttranslational modifications (PTMs) are implicated in the neuropathology of dementia [5] which are also known as degenerative protein modifications (DPMs) [6, 7]. Understanding the role(s) of DPMs is thus expected to provide novel insight on the pathogenesis of these aging-associated diseases, and it may aid on the development of new diagnostic and therapeutic tools. Two of these crucial DPMs that are thought to contribute to the neuropathology of dementia are citrullination and carbamylation [8–10]. Citrullination is an aging-associated DPM [11, 12] mediated by peptidylarginine deiminases (PADs), a calcium-dependent family of enzymes that generate the non-coded amino acid citrulline (Cit) from arginine (Arg) residues by hydrolysis [13]. Carbamylation of lysine (Lys) residues oppositely occurs spontaneously using the urea intermediate isocyanic acid as substrate to generate the non-coded amino acid homocitrulline (HCit) [10]. Cit and HCit are almost identical residues and differ only by one carbon in their side chain, although HCit results from a non-enzymatic chemical reaction and Cit formation is enzymatic [14]. Particularly, both non-coded amino acids are considered as ureido DPMs (uDPMs); hence, they share an ureido group in the side chain [14]. Citrullination is known to cause loss of positive charge in Arg residues, which in turn disturbs the structural order of the protein and makes it more prone to proteolysis [11]. Similarly, carbamylation neutralizes the positively charged Lys residues in an irreversible manner [15] and can result in altered function and pathogenic conformation [16, 17]. Cit and HCit, thus, favor protein denaturation and proteolysis and can also be recognized by the immune system as non-naturally coded amino acids [6, 7, 11, 18]. This former fact leads to the apparition of autoimmunity and inflammation, which are at the very basis of several chronic human diseases [19–21].

The neuropathology of dementia, including AD + CVD, is associated with dysfunctional proteins that form oligomeric structures in the brain parenchyma known as amyloids [6, 22]. Amyloids are resistant to proteolytic degradation and consequently sequestered into inclusion bodies, which are further compartmentalized in the brain parenchyma to reduce toxicity [23–25]. Although the role of protein inclusions appears mainly to be protective [23], chronic accumulation of these structures in the brain is likely to threaten neuronal function and viability [26]. Within the central nervous system (CNS), several DPMs have been associated with the formation and accumulation of amyloids [5] and some studies have suggested an association between citrullination, proteinopathy [27, 28], and brain vascular dysfunction [29, 30]. However, despite the implication that uDPMs might be involved in the neuropathology of AD + CVD, the extent of uDPMs in affected brain cells is unknown and the role(s) of citrullination and carbamylation in the neuropathology of dementia remain far to be fully elucidated [7]. Similarly, although uDPMs are associated with multiple sclerosis (MS) [20] and other autoimmune diseases [21], any apparent nexus between uDPMs and neuroinflammation in AD + CVD has yet to be defined.

The ability to identify and quantify citrullinated and carbamylated proteins in complex samples is essential to understand their role(s) in normal cellular processes and disease states. Though until now, very few uDPM-containing proteins have been identified and characterized from brain-diseased proteomes [31]. This is mainly due to the chemical and structural similarities that Arg and Lys present with their respective modified counterparts, and between Cit and HCit, which by the use of classical biochemical methods result in significant cross-reactivity [14, 21, 32]. Recent advances in liquid chromatography mass spectrometry (LC-MS/MS)-based proteomics have made possible the unbiased identification of proteins in complex samples and characterization of isoforms expression, turnover rate, subcellular localization, PTMs, and quantification of altered abundances in disease states [33]. Previously, we have adopted such approaches to characterize neurodegenerative proteomes and other conditions [6, 7, 34]. As part of these studies, we recently developed ultracentrifugation-electrostatic repulsion hydrophilic interaction chromatography (UC-ERLIC)-coupled mass spectrometry to isolate and characterize soluble and aggregated proteins from human brain tissues [35]. Here, we have used this approach to undertake a comparative characterization of uDPMs in the soluble and aggregated postmortem brain proteomes of AD + CVD and age-matched controls. Our results show the novel identification of relevant dementia-specific citrullinated and carbamylated brain proteins. This study thus provides new insight into the implications of uDPMs in the neuropathology of AD + CVD and will pave the way for further investigations on uDPMs as potential targets for therapeutic intervention or disease-state biomarkers in this fatal dementia subtype.

## Methods

### Chemicals and reagents

All the reagents used in this study were purchased from Sigma-Aldrich (St. Louis, MO, USA) unless otherwise specified. Protease inhibitor cocktail tablets were purchased from Roche (Basel, Switzerland), and sequencing-grade-modified trypsin was purchased from Promega (Madison, WI, USA).

### Postmortem brain tissues

AD + CVD and healthy age-matched control (Ctrl) autopsied human brain tissues (middle temporal lobe, BA21 region) were generously donated by the Harvard Brain Tissue Resource Center (HBTRC, Boston, MA, USA). All patients met clinical diagnosis for dementia and histological criteria for AD + CVD (mixed dementias) at the time of autopsy. The available details of the samples, which include age, postmortem delay, and gender can be found in Additional file 1: Table S1. Three biological replicates were independently analyzed in this study for each experimental condition (soluble and particulate proteomes). Brain tissues were stored in liquid nitrogen from the time of autopsy and subsequently kept at −150 °C until use. BA21 brain region was dissected, meninges were removed, and ~ 100 mg of tissue from each subject were washed thrice during 10 min in 1X PBS.

### Homogenization of postmortem brain tissues

Dissected brain tissues were homogenized as previously described [36], and all the following described procedures on postmortem tissues were performed on ice. Brain tissues were suspended in 250 μL of homogenization buffer (0.5% (*w*/*v*) sodium deoxycholate (SDC) in 100 mM ammonium acetate (AA) (pH 6.0) or 1% (*w*/*v*) *N*-lauroylsarcosine in 30 mM Tris-HCl (pH 7.4) and 150 mM NaCl and were supplemented with protease inhibitor cocktail. Approximately, 100 mg of previously washed metallic beads (0.9–2.0-mm particles) were added to each safe-lock tube that contained the tissues and homogenization buffer. Homogenization was then performed using the bullet blender homogenizer (Next Advance, NY, USA) at high intensity during 5 min at 4 °C. The tissue homogenates were subsequently centrifuged at 10,000 × *g*, 4 °C, for 10 min, and the supernatants were collected. Remaining pellets were subject to further rounds of homogenization till the pellet was not observable. The obtained supernatants from each round of homogenization were finally combined and briefly vortexed.

### Isolation of the soluble and particulate brain proteomes by UC-ERLIC

Soluble and particulate brain proteomes were isolated from brain homogenates by ultracentrifugation-electrostatic repulsion hydrophilic interaction chromatography (UC-ERLIC)-coupled mass spectrometry-based proteomics as we recently reported [35], although with minor modifications in this study. Soluble homogenates were centrifuged at 3000 × *g*, 4 °C, during 10 min to remove cell debris. Supernatants were then subjected to ultracentrifugation at 112,000 × *g*, 4 °C, during 60 min, transferred to a new tube, and subjected to two further rounds of ultracentrifugation under the same conditions. Supernatants obtained from each round of ultracentrifugation were combined and transferred to new tubes (soluble fraction). The generated particulate pellets from the last two ultracentrifugation rounds were re-solubilized in 150 μL of 5% SDC (*w*/*v*) in 100 mM AA or 3% sodium dodecyl sulfate (SDS) in 1X PBS when the sample was homogenized in sarcosyl and combined to obtain the final particulate proteome.

### In-solution tryptic digestion of human brain proteins

Soluble and particulate proteomes were subject to in-solution tryptic digestion as previously described [37]. Briefly, 0.5 mg of proteins from each sample and condition previously quantified by bicinchoninic acid protein assay (BCA) were reduced with 10 mM dithiothreitol (DTT) at 60 °C during 30 min. Proteins were alkylated with 20 mM iodoacetamide at room temperature protected from the light during 45 min. Subsequently, the samples were diluted 10-fold with 100 mM AA containing 10 mM DTT and incubated at 37 °C for 30 min. Digestion was performed at 30 °C overnight at 1:20 (*w*/*w*) enzyme-to-substrate ratio using sequencing-grade-modified trypsin. Enzymatic digestion was quenched by addition of 0.5% formic acid (FA). SDC under acidic conditions was pelleted by centrifugation at 12,000 × *g*, 4 °C, for 10 min as previously indicated [37]. The supernatant containing tryptic-digested peptides was then collected, and the SDC pellet was re-suspended in 0.5% ammonium hydroxide for further peptide recovery. SDC was again precipitated by the addition of 0.5% FA and pelleted by centrifugation at 12,000 × *g*, 4 °C, for 10 min. After centrifugation, the supernatant was collected. Recovery of peptides from the SDC pellet was performed thrice. All the supernatants were combined together. Tryptic-digested peptides were desalted using a C-18 Sep-pack 1 g cartridge (Waters, Milford, MA, USA). Elution was performed with 1 mL of 75% acetonitrile (ACN), 0.1% FA buffer. The eluted peptides were then dried using a vacuum concentrator (Eppendorf, Hamburg, Germany) and reconstituted for HPLC fractionation with 200 μL of 80% ACN, 0.1% acetic acid.

### In-gel digestion of human brain proteins

SDS-solubilized proteins from the sarcosyl particulate fractions were resolved in a 12% SDS-PAGE gel. PAGE gel was stained using coomassie brilliant blue, and protein lanes were divided in 12 main areas according to the observed protein intensities. Gel lanes were cut into approximately 1-mm^2^ cubes and washed with 25 mM ammonium bicarbonate (ABB) in 50% ACN. Peptides contained in distained gel pieces were reduced in 10 mM DTT in 25 mM ABB at 60 °C for 1 h and subsequently alkylated in 55 mM IAA in 25 mM ABB at room temperature for 45 min in the dark. The gel cubes were then dehydrated in 100% ACN in two washes for 1 min each and dried in the speedvac for 5 min at 30 °C. Tryptic digestion of proteins was performed overnight at 37 °C by the addition of 10 ng/μl of sequencing-grade-modified trypsin (prepared in 25 mM ABB buffer). Tryptic-digested peptides were extracted from the gel cubes with 50% ACN and 5% acetic acid by vigorous vortexing during 30 min. The extraction step was repeated four times, and the obtained supernatants were combined and dried in the speedvac. The dried peptides were then re-suspended in 3% ACN, 0.1% FA, for subsequent LC-MS/MS analysis.

### High-pressure liquid chromatography fractionation

Reconstituted tryptic peptides after in-solution digestion were fractionated by a PolyWAX column (4.6 × 200 mm, 3 μm, PolyLC, Columbia, MD, USA) using a Shimadzu Prominence UFLC system (Kyoto, Japan). Peptide intensities were monitored at 280 nm. Peptides were separated in a 72-min gradient using 80% ACN, 0.1% acetic acid, as mobile phase A, and 10% ACN, 0.1% FA, as mobile phase B. Separation of peptides was carried out in a 60-min gradient at a flow rate of 1 ml/min as follows: 0% for 5 min, 0–20% B for 25 min, 20–33% B for 10 min, 33–60% B for 10 min, and 60–100% B for 5 min, followed by 10 min at 100% B. Fractions were collected every minute, partially dried in a vacuum concentrator to reduce the volume, and pooled according to peak intensities. Combined fractions were dried completely and reconstituted in 3% ACN, 0.1% FA.

### Liquid chromatography mass spectrometry analysis

Analysis of peptides was performed using an Orbitrap Elite mass spectrometer coupled with a Dionex UltiMate 3000 UHPLC system from Thermo Fisher Scientific Inc. (Bremen, Germany). Approximately, 2 μg of peptides were injected into a reverse phase Acclaim PepMap RSL column (75 μm ID × 15 cm, 2-μm particle size, Thermo Fisher Scientific Inc.) maintained at 35 °C and using a flow rate of 0.3 μL/min. Peptides were separated in a 60-min gradient using 0.1% FA as mobile phase A and 90% ACN, 0.1% FA, as mobile phase B. The gradient used for the separation of peptides was as follows: starting at 3% mobile phase B for 1 min, 3–35% mobile phase B over 47 min, 35–50% mobile phase B over 4 min, and 50–80% mobile phase B in 6 s and maintained isocratic for 78 s. Then, reverted to initial conditions over 6 s and maintained isocratic for 6.5 min. Tryptic-digested peptides were sprayed using a Bruker-Michrom Inc. Michrom’s Thermo Captive Spray nanoelectrospray ion source (Bruker-Michrom Inc., Auburn, CA, USA) with 1.5 kV spray voltage. Orbitrap Elite mass spectrometer was set to positive mode for data acquisition using Xcalibur 2.2 SP1.48 software (Thermo Fisher Scientific Inc., Bremen, Germany). Data acquisition was performed by alternating between full Fourier transform-mass spectrometry (FT-MS; 350–2000 m/z, resolution 60,000, 1 μscan per spectrum) and FT-MS/MS (150–2000 m/z, resolution 30,000, 1 μscan per spectrum) for the ten most intense ions above 500 count threshold with charge > + 2. Fragmentation was performed using high-energy collisional dissociation (HCD) mode with 32% normalized collision energy. Automatic gain control values for FT-MS and FT-MS/MS were set at 1 × 10^6^.

### Bioinformatics and data analysis

LC-MS/MS data was searched in PEAKS Studio version 7.5 [38] (Bioinformatics Solutions, Waterloo, Canada). Precursor ion tolerance and fragment ion tolerance were kept during the database search at 10 ppm and 0.05 Da, respectively. Trypsin with non-specific cleavage on both ends of the peptide was allowed. Carbamidomethylation of cysteine residues was considered as fixed modification. The PEAKS PTM algorithm [39] was used to identify the presence of uDPMs in the brain proteomes. False discovery rate was kept as default by the software, and decoy-target identification of the peptides was performed. To extract the most abundant peptides from the particulate brain proteomes, only those peptides with relative signal intensities higher or equal to 10^8^ were considered. This restrictive criterion allowed for the unique consideration of the most confidently identified peptides. The mean signal intensity was calculated individually for every subject and experimental condition (soluble and particulate brain proteomes) and contributed to the calculation of the grand mean signal intensity for each group, which has been reported here.

Enrichment distribution of the AD + CVD particulate brain proteomes was analyzed using the enrichment analysis software FunRich [40] as described [41]. The absolute number of unique proteins with identified citrullinated (Arg) and carbamylated (Lys) residues was included in this analysis. To analyze the aggregation pattern of the identified neuroinflammatory markers in AD + CVD only the proteins that were consistently identified in at least two individuals of the same group were considered. Student’s *t* test was performed to establish statistical significance between experimental groups (*p* < 0.05). All statistical analyses and graphs were performed and plotted in GraphPad Prism v6.01 (La Jolla, CA, USA). The bioinformatics study of the biochemical composition of hypercitrullinated proteins in the brain proteome of AD + CVD was performed using an in-house-created macro for Microsoft Excel. All the possible citrulline triplets were screened in this analysis resulting in a total of 400 combinations as shown in Additional file 2: Dataset 1. The total signal intensity for every group of peptides triplet was then ranked from the highest to lowest. Only those top five triplets that were consistently identified as hypercitrullinated in all subjects and conditions are reported. Data is reported as mean ± standard deviation (SD).

## Results

### Composition of brain amyloids in AD + CVD

Sarcosyl can be considered as a gold standard detergent for biochemical characterization of insoluble aggregates from brain tissues [42, 43]. However, the use of this detergent has to be coupled to in-gel digestion. To characterize the molecular composition of brain amyloids, we proposed the use of sodium deoxycholate (SDC), a detergent that allowed in-solution digestion and multidimensional chromatography, to improve the identification of posttranslationally modified peptides [37]. For confirmatory purposes, we performed a comparative study to analyze the performance of both partitioning methodologies. No substantial differences were observed on the total amount of amyloidal protein obtained by sarcosyl and SDC in BCA assays (1.91 mg vs. 1.94 mg, respectively). Similarly, no obvious differences were observed on the total number of proteins identified by LC-MS/MS from both methodologies (Additional file 3 **:** Figure S1a and Additional file 4: Dataset 2), although as expected, the SDC condition increased by near 50% the identification of unique uDPM-containing peptides (Additional file 3: Figure S1b).

**Fig. 1:**
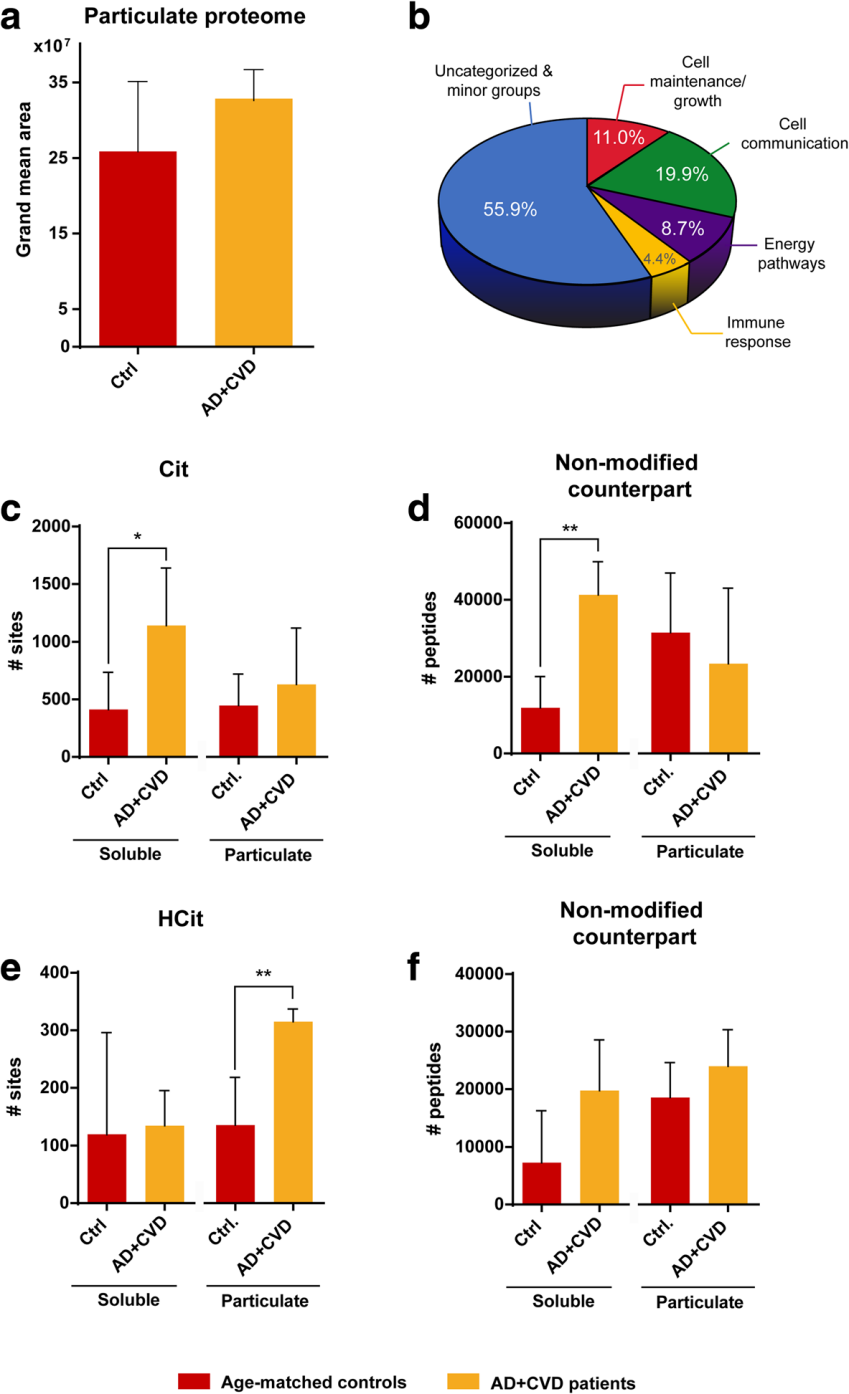
Profiling of the soluble and particulate brain proteomes in AD + CVD. **a** Grand mean ion intensity of the most abundant peptides from the AD + CVD and age-matched control particulate proteomes. **b** Enrichment analysis of the dementia-specific proteins identified in the particulate proteomes of AD + CVD. **c** Distribution of citrulline sites in the soluble and particulate proteomes of AD + CVD and age-matched controls. **d** Distribution of citrulline non-modified counterpart peptides in the soluble and particulate proteomes of AD + CVD and age-matched controls. **e** Distribution of homocitrulline sites in the soluble and particulate proteomes of AD + CVD and age-matched controls. **f** Distribution of homocitrulline non-modified counterpart peptides in the soluble and particulate proteomes of AD + CVD and age-matched controls. Data is reported as mean + SD (*p < 0.05 one-tailed Student’s *t* test. **p < 0.05 two-tailed Student’s *t* test)

A similar number of proteins were identified by UC-ERLIC in the particulate proteomes of age-matched controls and AD + CVD (Additional file 5: Dataset 3). However, careful analysis of the most abundant peptides has shown apparently higher accumulation of dysfunctional proteins in the particulate proteomes of the dementia brains (Fig. 1a). Screening of those particulate proteins uniquely found in AD + CVD revealed cell communication (synaptic proteins) and energy production (mitochondrial proteins) as the two most abundant protein categories that become aggregated in these tissues (Fig. 1b). Cell maintenance and immune response though were also found in that analysis as meaningful abundant protein categories in the particulate proteomes of AD + CVD (Fig. 1b). In particular, immune response-related proteins represent near the 5% of the total unique aggregated proteins in AD + CVD brains (Fig. 1b).

### uDPMs and brain proteinopathy

An increase in the formation of Cit and HCit residues was observed in the brains of AD + CVD compared to those of age-matched controls (Fig. 1c, e). Nevertheless, higher amount of Cit residues was uniquely observed in the soluble brain fractions of dementia brains (Fig. 1c). Analysis of unmodified proteins prone to contain Cit residues also indicated a relative increase of these proteins in the soluble brain proteomes of AD + CVD (Fig. 1d). Contrary, higher amount of HCit residues was uniquely observed in the particulate fractions of dementia brains (Fig. 1e). No significant differences were observed on the aggregation pattern of the unmodified proteins prone to contain HCit residues (Fig. 1f). However, sequestration of unmodified proteins prone to contain Cit residues to the particulate brain fraction was a meaningful phenomenon apparently characteristic of non-demented brains (Fig. 1d).

We then investigated group-specific uDPMs, considering uniquely those modified residues that are consistently modified in at least two individuals of the same group and that at the same time were not identified in the same experimental condition (soluble and particulate) in any individual of the other group. A total of seven specific HCit residues were confidently identified in the particulate fraction in this screening (Table 1). These dementia-specific HCit-containing proteins include brain acid soluble protein 1 (BASP1), spectrin alpha chain 1 (SPTAN1), and glial fibrillary acidic protein (GFAP) (Table 1). Stoichiometric analysis of dementia-specific HCit residues revealed that the frequency of modification of Lys residues exceeded the 50% in the majority of these proteins (Table 1). The whole list of homocitrulline sites identified in the particulate brain proteomes of AD + CVD is included in Additional file 6: Dataset 4.

**Table 1:** List of proteins that contain exclusive HCit residues in the particulate brain proteome of AD + CVD

Gene	Protein	Peptide	Protein residue^b^	Secondary Structure^c^	Stoichiometry^d^
SPTAN1	Spectrin alpha chain non-erythrocytic 1	K^a^HQLLEADISAHEDR	1699	–	50
ATP1A3	Sodium/potassium-transporting ATPase subunit alpha-3	K^a^ADIGVAMGIAGSDVSK	717	–	16
ACTB	Actin cytoplasmic 1	NSIMK^a^CDVDIR	284	–	67
ATP6V0A1	V-type proton ATPase 116 kDa subunit a	K^a^ANIPIMDTGENPEVPFPR	74	–	22
ATP2B4	Plasma membrane calcium-transporting ATPase 4	K^a^ADVGFAMGIAGTDVAK	795	–	41
BASP1	Brain acid soluble protein 1	K^a^TEAPAAPAAQETK	150	–	50
GFAP	Glial fibrillary acidic protein	SK^a^FADLTDAAAR	260	Coil 2B	55

^a^The homocitrulline residue in the identified peptide

^b^The residue number in the protein sequence

^c^When available, the location of the HCit residue in the secondary structure of the protein

^d^The stoichiometric ratio (percentage of occurrence) of HCit from the total peptides identified that contain the Lys site in the protein (unmodified + modified)

### Citrullination and neuroinflammation

Several brain proteins that have been vastly implicated in autoimmunity and pro-inflammatory processes in a wide range of human diseases were identified in this study as containing dementia-specific pro-immunogenic Cit sites in the soluble brain proteome of AD + CVD subjects (Table 2). Stoichiometric analysis of these pro-immunogenic sites indicated that the frequency of modification of Arg residues was 100% for the vast majority of the specific sites identified (Table 2). The list of dementia-specific Cit sites that were identified in the soluble fraction of AD + CVD brains is shown in Additional file 7: Dataset 5. Of note, this list shows the identification of several histone molecules which contain Cit sites while being present in soluble form in the brain of dementia patients. The complete list of all identified soluble AD + CVD Cit sites, including those residues that are non-dementia specific is listed in Additional file 8: Dataset 6.

**Table 2:** List of proinflammatory and immunogenic proteins that contain exclusive Cit residues in the soluble brain proteome of AD + CVD. The proteins listed in this table have been previously implicated in inflammatory and autoimmune processes (reference(s) column)

Gene	Protein	Reference(s)	Peptide(s)	Protein residue(s)	Location	Stoicho-metry^a^
CNP	2′ 3′-cyclic-nucleotide 3′-phosp.	Birnbaum et al. 1996 [61]	ELR^b^QFVPGDEPR	224	Helix (223–226)	100
GFAP	Glial fibrillary acidic protein	Sasaki et al. 2014 [62]	SNLQIR^b^ETSLDTK	390	Tail (378–432)	100
INA	Alpha-internexin	Lu et al. 2010 [63]	RRPPASDGLDLSQAAAR^b^TNEYK	83	Head (1–87)	56
			KVFGDGSR^b^LSAR	28	Head (1–87)	100
HK1	Hexokinase-1	Norman et al. 2015 [64]	ITPELLTR^b^GK	331	N/A	100
			NGLSR^b^DFNPTATVK	53	Turn (53–55)	100
NEFL	Neurofilament light polypeptide	Jain et al. 2009 [65]	YVETPR^b^VHISS	23	Head (2–92)	100
ADD1	Adducin 1 (Alpha)	Rötzer et al. 2014 [66]	EDGHR^b^TSTSAVPNLFVPLNTNPK	479	–	67
RTN3	Reticulon-3	Chiurchiù et al. 2014 [67]	TQIDHYVGIAR^b^DQTK	1009	–	100
AK1	Adenylate kinase isoenzyme 1	Tüzün et al. 2007 [68]	YGYTHLSTGDLLR^b^SEVSSGSAR	44	Helix (39–48)	100
TAGL3	Transgelin-3	Na et al. 2015 [69]	R^b^GFSEEQLR	160	–	100
SYT7	Synaptotagmin-7	Chakrabarti et al. 2003 [70]	YKNSLETVGTPDSGR^b^GR	63	–	100
STMN1	Stathmin	Bsibsi et al. 2010 [71]	R^b^ASGQAFELILSPR	14	–	71
P2RX7	P2X purinoceptor 7	Lister et al. 2007 [52]	LPLALHDTPPIPGQPEEIQLLR^b^K	463	–	57
HSP90AA1	Heat shock protein HSP 90-alpha	Tukaj et al. 2016 [72]	GVVDSEDLPLNISR^b^EMLQQSK	400	–	40
NEUM	Neuromodulin	Hung et al. 2016 [73]	IQASFR^b^GHITR	43	–	100
MBP	Myelin basic protein	Zierath et al. 2015 [74]	FGYGGR^b^ASDYK	64	–	45
			SHGR^b^TQDENPVVHFF	213^c^	EAE (179–222)	41
			HGFLPR^b^HRDTGILDSIGR	165	–	32
			FFGGDR^b^GAPKR	183^c^	EAE (179–222)	75
			YLATASTMDHAR^b^HG	159	–	58
			FSWGAEGQRPGFGYGGR^b^ASDYKSAHK	264	–	45
CTTN	Src substrate cortactin	Labrador-Horrillo et al. 2014 [75]	HCSQVDSVR^b^GFGGK	119	–	100

^b^Indicates the citrulline residues in the identified peptides

^c^Indicates the MBP citrulline residues in mice that were identified in the protein homolog region that induces experimental autoimmune encephalomyelitis (EAE)

^a^Indicates the stoichiometric ratio (percentage of occurrence) of Cit from the total peptides identified that contain the Arg site in the protein (unmodified + modified)

Since apparently higher accumulation of unmodified proteins that are prone to contain Cit residues into the particulate fraction was observed in the brain of age-matched controls, we also scrutinized which AD + CVD citrulline residues become consistently aggregated in age-matched controls and remain soluble in the brain of dementia subjects during AD + CVD. We found that the citrulline residue 213 (SHGR^*^TQDENPVVHFF) of myelin basic protein (MBP), citrulline residues 24 (KVFGDGSR^*^LSAR) and 83 (RRPPASDGLDLSQAAAR^*^TNEYK) of alpha-internexin, and the citrulline residue 83 (IIPR^*^HLQLAIRNDEELNK) of histone H2AX were successfully identified in this analysis.

**Fig. 2:**
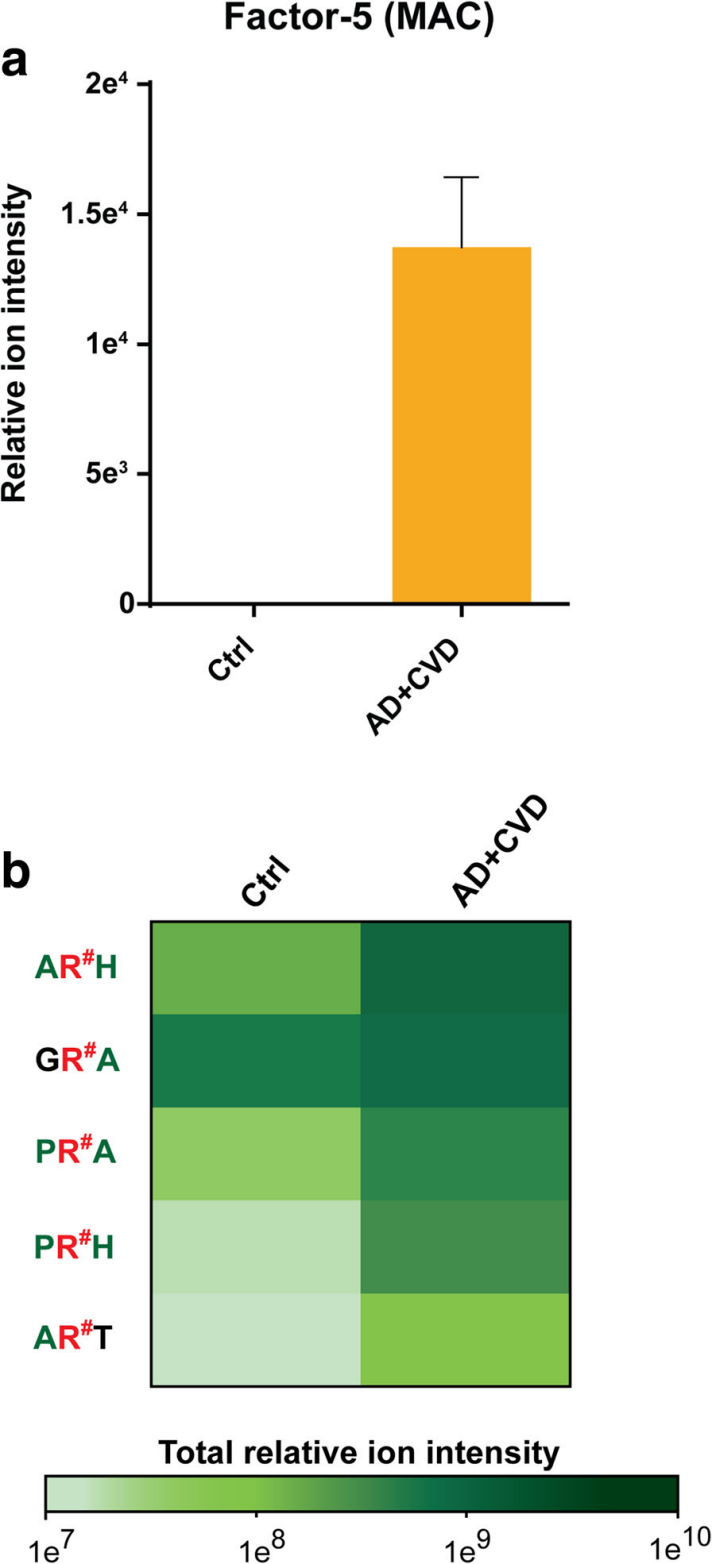
Citrullination and neuroinflammation in the brain proteome of AD + CVD. **a** MS precursor ion intensity of the neuroinflammatory marker complement-5 core member of the membrane attack response. This marker was only identified in soluble form in the brain proteomes of AD + CVD, and it was the unique neuroinflammatory marker identified in association with the observed aggregation pattern of Cit residues. **b** Heatmap showing the levels of the top five most intense Cit amino acid triplets in the brain proteome of AD + CVD and age-matched controls. The small residue alanine (Ala) and the aromatic residues proline (Pro) and histidine (His) are commonly found at the flanks of Cit residues in the most abundant citrullinated peptides of the brain proteome. The column of the amino acid triplets in green color indicates the common top five amino acids and in black color, the uncommon amino acids

Soluble hypercitrullinated proteins are associated with enhanced autoimmunity and consequent neuroinflammation, and to further explore this hypothesis in AD + CVD, we evaluated the pattern of aggregation of the identified neuroinflammatory markers from all the profiled brain tissues. We observed that the protein complement factor-5, an essential member of the membrane attack complex (MAC), was the unique neuroinflammatory marker found in association with Cit residues in the soluble brain fraction of AD + CVD subjects (Fig. 2a).

### Biochemical characterization of increased citrullination in the brain proteome

In order to elucidate if enzymatic citrullination of susceptible brain proteins is a randomized calcium-dependent process or conversely it proceeds by any organized and specific biochemical pattern, we scrutinized here the identified amino acid triplets that surround citrulline residues in all the profiled brain proteomes. We strikingly observed that the most abundant citrullinated Arg residues were commonly surrounded by either the small amino acid alanine (Ala) or the aromatic amino acids proline (Pro) and histidine (His) as shown in Fig. 2b.

## Discussion

Unbiased profiling of the soluble and particulate brain proteomes confirmed that formation of amyloids in the brain parenchyma is an aging-associated process that takes place with independence of the apparition of dementia symptoms. Higher accumulation of proteins was though observed in the brains of dementia subjects. These findings were consistent with previous reports showing that there is basal amyloid formation in the brain parenchyma of aged subjects [44] and higher accumulation of dysfunctional proteins in brain tissues undergoing neurodegeneration [35, 45]. Detailed analysis of the particulate proteomes has shown immune response-related proteins as a protein category that becomes preferentially aggregated in the temporal lobe of dementia subjects. Although neuroinflammation is considered a factor that plays a causal role in the development of dementia [46, 47], the existing links between proteinopathy and neuroinflammation in this progressive and fatal disease remain poorly understood [48].

Increase in uDPMs, as identified in dementia brains, suggests activation of a defective immune and pro-inflammatory response [48]. uDPMs are associated with apparition of inflammation and proteinopathy; however, only very few proteins that might mediate these pathogenic outcomes in dementia have been until now identified [27, 28]. We previously reported that an increase in citrullination in certain MBP residues did not apparently influence the aggregation ability of the protein [49]. Here, we globally confirm this finding as citrullinated proteins tended to remain soluble in the temporal lobe of AD + CVD subjects. In further detail, we found that the purinergic receptor P2X7 was modified by a dementia-specific Cit residue in the soluble brain proteome of AD + CVD. To the best of our knowledge, this is the first time that this crucial immune receptor appears modified by PADs in a dementia-relevant manner. Activation of the immune response in the brain is an ATP-dependent process initiated by injury and death of the surrounding cells [50, 51] and mediated by P2X7 in microglia [52–54], a receptor that also induces increase in citrullination and release of Cit residues out of the cell [55]. Similarly, several dementia-specific Cit sites were identified in histones H2 and H4 in the soluble brain fractions, which suggests ongoing formation of neurotrophil extracellular traps (NETs). NETs have been proven as molecular culprits in the pathogenesis of rheumatoid arthritis (RA) and other autoimmune diseases [56–59]. Formation of NETs is mediated by citrullination of histones, which neutralizes the charge of these molecules promoting chromatin decondensation [60]. Besides, the following proteins CNP, GFAP, Alpha-internexin, Hexokinase-1, Neurofilament Light Polypeptide, Adduccin-1, Reticulon-3, Adenylate kinase isoenzyme 1, Transgelin-3 Synaptotagmin-7, Stathmin, P2X purinoceptor 7, Heat shock protein HSP 90-alpha, Neuromodulin, MBP, and Cortactin were also identified containing dementia-specific Cit residues in the soluble brain proteome of AD + CVD. Considerably, all these modified proteins have been previously implicated in autoimmunity and inflammation in several human diseases [61–75], though many of them were not previously identified in the sphere of dementia. An increase in citrulline in soluble brain fractions was associated with the increase of the MAC protein complement factor-5 in AD + CVD, the unique neuroinflammatory marker found upregulated in the soluble fraction of dementia subjects compared to age-matched controls. We also observed that an increase in citrullination in AD + CVD brains was favored by the small amino acid Ala and the aromatic amino acids Pro and His at the flank of Arg residues. The biochemical patterns that influence aberrant citrullination in dementia hold great promise in the search for novel pharmacological targets to fight chronic neuroinflammation in this disease.

Our main aim for this study was also to understand how uDPMs can influence the aggregation ability of temporal lobe proteome in AD + CVD. We observed that HCit, a spontaneous non-enzymatic aging-associated uDPM that becomes hardly distinguishable from Cit by conventional biochemical methods [14, 32, 76], seems to clearly contribute to brain proteinopathy in this dementia subtype. The observed enrichment of HCit dementia-specific sites into the particulate fraction of AD + CVD brains may indicate involvement of HCit in early brain inflammatory processes. Mydel and colleagues demonstrated that HCit triggered early activation of proinflammatory cells, which was the primary step to recognition of Cit residues in autoimmune inflammation [77]. Besides, Currais and colleagues [78] recently suggested that certain proteins from the particulate brain fraction may contribute to the progression of neuroinflammation during dementia. In line with this hypothesis, we highlight the proinflammatory proteins GFAP and BASP1 which appear as good candidates for further research in this direction. These proteins were identified in this study as containing dementia-specific HCit residues in the particulate brain fraction of dementia subjects. Other proteins identified with exclusive AD + CVD particulate HCit sites are alpha-spectrin, sodium/potassium ATPase, actin 1, V-type proton ATPase 3, and calcium-transporting ATPase 4. Gorisse and colleagues suggested that although HCit-containing proteins should be degraded by common proteolytic mechanisms, some of these proteins seem to show surprising slow turnover rates even when the protein quality control systems in the cells are functional [10]. Similarly, HCit is known to promote endothelial dysfunction and vascular disease [79]; however, uncovering its specific contributions to the pathology of the neurovascular unit in mixed dementias requires of further research.

## Conclusions

Citrullination and carbamylation are involved in the neuropathology of AD + CVD through different means. Increase in citrullination involved apparition of exclusive dementia Cit residues in pro-immunogenic proteins, which was associated with pathogenic neuroinflammation. We also observed how proteins prone to contain citrulline residues in the brain proteome of dementia subjects remain soluble whereas these proteins in the brain of age-matched controls tend to be sequestered into aggregates. Protein carbamylation was associated with brain proteinopathy and accumulation of insoluble proteins in the brain parenchyma of AD + CVD. Altogether, these findings provide relevant and novel insight on the biochemical processes triggered by uDPMs to contribute to the neuropathology of mixed dementias.

## Additional files

Additional file 1: Table S1. Detail of the human postmortem brain tissues analyzed. (DOC 34 kb)

Additional file 2: Dataset 1.1: Total group LC-MS/MS peak intensity of the peptides that contain the citrulline triplet in the BA21 brain proteome. (XLS 176 kb)

Additional file 3: Figure S1. Comparative analysis of soluble and particulate partitioning methodologies by sarcosyl coupled to in-gel digestion and sodium deoxycholate (SDC) coupled to in-solution digestion. a. Number of total proteins identified by LC-MS/MS in the particulate brain fractions from both methodologies. b. Number of unique peptides that contain uDPM sites in the LC-MS/MS analyzed particulate brain fractions from both methodologies. (TIFF 212 kb)

Additional file 4: Dataset 2. List of total proteins identified in the particulate brain fraction of AD + CVD by sarcosyl coupled to in-gel digestion. (XLS 1311 kb)

Additional file 5: Dataset 3. List of proteins identified in the soluble and particulate brain fractions. (XLS 6489 kb)

Additional file 6: Dataset 3. List of homocitrulline sites identified in the particulate brain proteomes of AD + CVD. (XLS 68 kb)

Additional file 7: Dataset 4. Complete list of dementia-specific citrulline sites identified in the soluble brain proteome of AD + CVD. (XLS 82 kb)

Additional file 8: Dataset 5. List of all identified citrulline sites in the soluble brain proteome of AD + CVD. (XLS 194 kb)

## Abbreviations

AA: Ammonium acetate
ABB: Ammonium bicarbonate
ACN: Acetonitrile
AD + CVD: Alzheimer’s disease with cerebrovascular disease
Arg: Arginine
BASP1: Brain acid soluble protein 1
BCA: Bicinchoninic acid assay
CNP: 2′,3′-Cyclic-nucleotide 3′-phosphodiesterase
DPMs: Degenerative protein modifications
DTT: Dithiothreitol
FA: Formic acid
GFAP: Glial fibrillary acidic protein
His: Histidine
HPLC: High-pressure liquid chromatography
LC-MS/MS: Liquid chromatography-coupled mass spectrometry
Lys: Lysine
MAC: Membrane attack complex
MBP: Myelin basic protein
P2X7: Purinergic receptor P2X7
PADs: Peptydilarginine deiminases
Pro: Proline
RA: Rheumatoid arthritis
SDC: Sodium deoxycholate
SPTAN1: Spectrin alpha chain 1
UC-ERLIC: Ultracentrifugation-electrostatic repulsion hydrophilic interaction chromatography-coupled mass spectrometry
uDPMs: Degenerative protein modifications that contain a ureido group
