# Supplementary material for: Brain ureido degenerative protein modifications are associated with neuroinflammation and proteinopathy in Alzheimer’s disease with cerebrovascular disease
Source: J Neuroinflammation. 2017 Sep 2;14:175. doi: 10.1186/s12974-017-0946-y (PMC5581431; doi:10.1186/s12974-017-0946-y)
Supplement: Supplementary file 1 — Detail of the human postmortem brain tissues analyzed. (DOC 34 kb) [file 12974_2017_946_MOESM1_ESM.doc]

**Supplementary Table 1.** Detail of the human post-mortem brain tissues analyzed.

|  | **Age** | **Gender** | **PMD** | **Braak** |
| --- | --- | --- | --- | --- |
| Dementia 1 | 65 | M | 7.8 | - |
| Dementia 2 | 68 | F | 18.2 | - |
| Dementia 3 | 63 | M | 30.5 | - |
| Age-matched Ctrl. 1 | 69 | M | 18.4 | 1 |
| Age-matched Ctrl. 2 | 62 | M | 21.4 | 1 |
| Age-matched Ctrl. 3 | 67 | M | 23.5 | 1 |
