# Supplementary figures and images for: Brain ureido degenerative protein modifications are associated with neuroinflammation and proteinopathy in Alzheimer’s disease with cerebrovascular disease
Source: J Neuroinflammation. 2017 Sep 2;14:175. doi: 10.1186/s12974-017-0946-y (PMC5581431; doi:10.1186/s12974-017-0946-y)

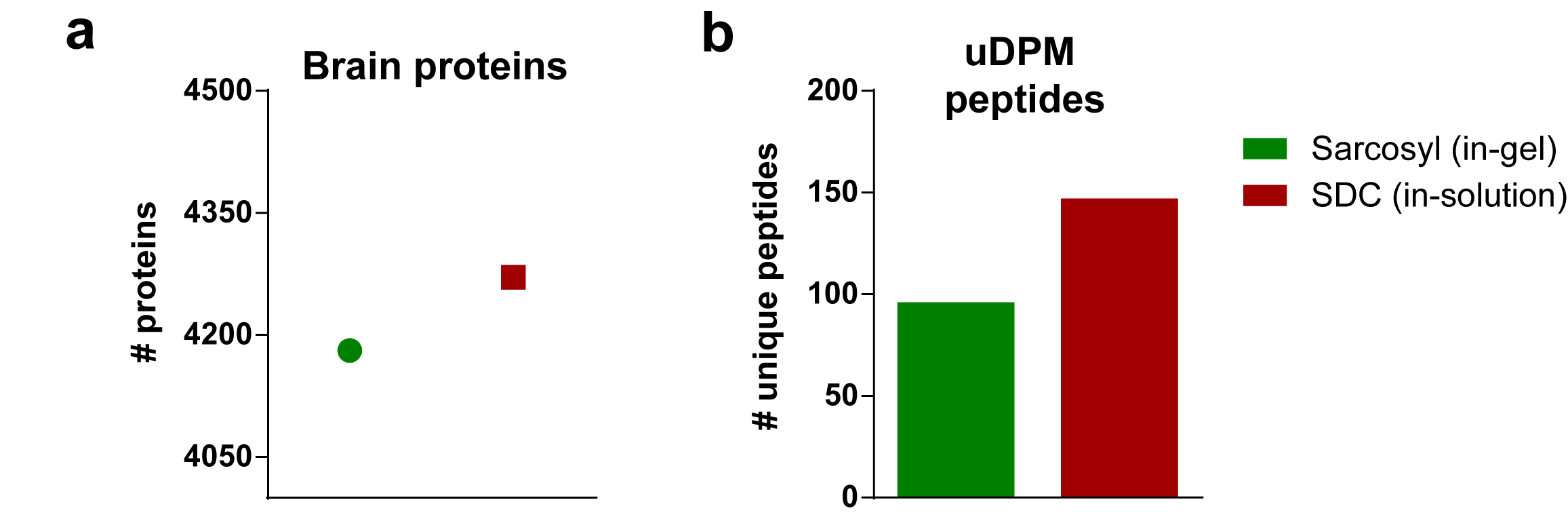

Supplement: Supplementary file 3 — Comparative analysis of soluble and particulate partitioning methodologies by sarcosyl coupled to in-gel digestion and sodium deoxycholate (SDC) coupled to in-solution digestion. a. Number of total proteins identified by LC-MS/MS in the particulate brain fractions from both methodologies. b. Number of unique peptides that contain uDPM sites in the LC-MS/MS analyzed particulate brain fractions from both methodologies. (TIFF 212 kb) [file 12974_2017_946_MOESM3_ESM.tif]
